# The Multi-Faceted Utility of Cardiovascular Magnetic Resonance Imaging: Editorial on Special Issue “Advances in Cardiovascular Magnetic Resonance”

**DOI:** 10.3390/diagnostics13233501

**Published:** 2023-11-22

**Authors:** Minjie Lu, Arlene Sirajuddin

**Affiliations:** 1 Department of Magnetic Resonance Imaging, Fuwai Hospital, Chinese Academy of Medical Sciences & Peking Union Medical College, Beijing 100037, China; 2 Key Laboratory of Cardiovascular Imaging, Chinese Academy of Medical Sciences, Beijing 100037, China; 3 Department of Health and Human Services, National Heart, Lung and Blood Institute, National Institutes of Health, Bethesda, MD 20892, USA

Cardiovascular magnetic resonance (CMR) imaging has emerged as a versatile tool for evaluating and managing a variety of cardiovascular diseases. In this Special Issue, “Advances in Cardiovascular Magnetic Resonance”, a total of 14 articles (11 original research articles, 1 case report, and 2 comprehensive reviews) demonstrated various advances in the clinical applications of CMR imaging. These studies highlighted the efficacy of CMR imaging in characterizing idiopathic dilated cardiomyopathy (IDCM), diagnosing Loeffler endocarditis, evaluating cardiac function in males with acquired immune deficiency syndrome (AIDS), predicting left ventricular remodeling (LVR) following primary percutaneous coronary intervention (PPCI), and assessing the risk of major adverse cardiovascular events (MACE) in patients with left ventricular noncompaction (LVNC). Additionally, CMR imaging techniques, such as tissue-tagging (TT-CMR) and feature-tracking CMR (FT-CMR), showed promising results in determining cardiac deformation and functional dynamic geometry parameters.

## 1. Ischemic Heart Disease

The burden of ischemic heart disease remains significant, both in terms of its prevalence and its impact on individuals and society. According to the World Health Organization (WHO), ischemic heart disease is the leading cause of death worldwide, and it accounted for more than 8 million deaths in 2019 [1].

Ma et al. focused on exploring predictive parameters for LVR following PPCI in patients with acute anterior myocardial infarction (AAMI) using CMR imaging [2]. This study found that both global and regional CMR parameters were valuable in predicting LVR in AAMI patients following PPCI, with the local parameters of the infarct zones found to be superior to those of the global ones.

Palumbo et al. evaluated the role of stress perfusion cardiac magnetic resonance (spCMR) in predicting the risk of major cardiac events in patients with long-standing chronic coronary syndrome (CCS) and ischemia [3]. They included 35 patients who underwent coronary CT angiography (CCTA) and additional adenosine spCMR and the primary outcomes measured were heart failure and all major cardiac events. They concluded that spCMR modeling, including perfusion and strain anomalies, could be a powerful tool in predicting the risk of major cardiac events in patients with long-standing CCS, even in the absence of conventional imaging predictors.

## 2. IDCM

IDCM is a type of myocardial disease of unknown origin, with many cases thought to be genetic in nature. This condition can lead to heart failure, arrhythmias, and sudden cardiac death. Due to the lack of a clear cause, diagnosis and management of IDCM can be challenging.

The study by Tsabedze et al. investigated the use of CMR imaging in the characterization of IDCM in a cohort of patients in southern Africa who were suspected of having a genetic cause of their cardiomyopathy [4]. The study found that late gadolinium enhancement (LGE) was present in the majority of the participants and that mid-wall LGE enhancement was the most common pattern observed. The study also found that patients with LGE on CMR imaging had a lower risk of death than those without. These findings highlight the potential of CMR imaging for improving not only the diagnosis but also the management of IDCM in sub-Saharan Africa.

Zhang et al. investigated the expression of microRNAs (miRNAs) in myocardial tissues of hypertrophic cardiomyopathy (HCM) patients and found that some of the miRNAs had significant correlations with cardiac function and myocardial fibrosis, indicating their potential as biomarkers for left ventricular hypertrophy, fibrosis, and remodeling [5]. These findings suggest that miRNA levels in myocardial tissues could serve as potential biomarkers for HCM, potentially aiding in the early diagnosis and treatment of this disease.

Huang evaluated the role of LGE in predicting MACE in patients with LVNC and found that a specific ring-like pattern of LGE, free-wall, or mid-wall LGE, and LGE extent greater than 7.5% was associated with an increased risk of MACE [6]. This study suggests that risk stratification based on LGE may have value in predicting MACE in patients with LVNC.

Revnic et al. focused on the use of CMR imaging to detect myocardial replacement fibrosis in patients with nonischemic dilated cardiomyopathy (NIDCM) and sought to evaluate the association between collagen turnover biomarkers and replacement myocardial scarring by CMR and test their ability to predict outcomes in conjunction with LGE in patients with NIDCM [7]. They found that galectin-3 (Gal3), procollagen type I carboxy-terminal pro-peptide (PICP), and N-terminal pro-peptide of procollagen type III (PIIINP) were significantly increased in LGE+ individuals and were directly correlated with LGE mass. These circulating collagen turnover biomarkers could significantly predict cardiovascular outcomes, and their joint use with LGE could improve outcome prediction in patients with NIDCM. Overall, this study highlights the potential of using biomarkers in conjunction with CMR to detect myocardial fibrosis and predict outcomes in patients with NIDCM.

Brendel et al. evaluated the diagnostic performance of dark-blood LGE compared to that of conventional bright-blood LGE in detecting non-ischemic myocardial scarring [8]. The study included 343 patients with suspected non-ischemic cardiomyopathy who underwent both dark-blood and bright-blood LGE imaging. The results showed that dark-blood LGE had a sensitivity of 99%, a specificity of 99%, and an accuracy of 99% for detecting non-ischemic scarring, with no significant difference in scar size compared to bright-blood LGE. The study concludes that dark-blood LGE imaging is non-inferior to bright-blood LGE imaging in detecting non-ischemic scarring, suggesting it may be an equivalent method for detecting both ischemic and non-ischemic scars.

## 3. Inflammatory Cardiomyopathy

Inflammatory cardiomyopathy is a myocardial disease caused by inflammation that can result from several factors, including viral infections, autoimmune diseases, and drug reactions. Early diagnosis and proper management are critical to prevent severe complications and enhance patient outcomes.

Lupu et al. reported a case of Loeffler endocarditis in a patient presenting with heart failure with preserved ejection fraction (HFpEF) [9]. This case highlights the importance of early diagnosis and prompt management of Loeffler endocarditis in order to prevent serious complications and improve patient outcomes. CMR imaging was able to detect eosinophil and lymphocyte infiltration of the endomyocardium, as well as the formation of thrombus and fibrosis, leading to the diagnosis of Loeffler endocarditis.

Hou et al. evaluated the cardiac function of antiretroviral therapy-treated (ART-treated) males with AIDS using CMR imaging and found that those with a short disease duration may not develop obvious cardiac dysfunction as evaluated by routine CMR [10]. The findings suggest that routine CMR may not be necessary for ART-treated males with AIDS with a short disease duration, but it is important to consider individual patient characteristics and other factors when determining follow-up intervals.

## 4. MR Contrast Related and Extra-Cardiac Disease

In this issue, we feature two special research articles. The first study examines the relationship between MR contrast and nephrogenic systemic fibrosis (NSF). The second explores myocardial impairment associated with extra-cardiac disorders.

Gallo-Bernal et al. provide a comprehensive review of the existing evidence on the use of gadolinium-based contrast agents in cardiac imaging and the risk of NSF in patients with chronic kidney disease [11]. They highlight the importance of understanding the clinical characteristics and risk factors of NSF in order to prevent and recognize it as well as summarize the pathophysiology, clinical manifestations, diagnosis, and prevention of NSF related to the use of gadolinium-based contrast agents.

Yang et al. focused on the use of CMR imaging to examine the temporal changes in the cardiac cycle of patients with pulmonary hypertension (PH), a condition that alters the biventricular shape and temporal phases of the cardiac cycle [12]. They found that only the right ventricular ejection fraction (RVEF) was decreased in the ventricular function of the interventricular septal (IVS) non-displacement (IVS_ND_) group, and no temporal change in the cardiac cycle was found. In contrast, a prolonged isovolumetric relaxation time (IRT) and shortened filling time (FT) in both ventricles, along with biventricular dysfunction, were detected in the IVS displacement (IVS_D_) group. The IRT of the right ventricle (IRTRV) and the FT of the right ventricle (FTRV) in PH patients were associated with pulmonary vascular resistance, right cardiac index, and IVS curvature, and the IRTRV was also associated with the RVEF in a multivariate regression analysis. The researchers concluded that the temporal changes in the cardiac cycle were related to IVS displacement and mainly impacted the diastolic period of the two ventricles in the PH patients. Both IRT and FT changes may provide useful pathophysiological information on the progression of PH. The study suggests that CMR imaging can be a useful tool for understanding cardiac dysfunction in PH patients and for risk stratification of PH patients.

## 5. CMR Emerging Techniques

CMR has evolved significantly over the years, with the development of emerging techniques that have expanded its role in clinical practice. Among them, myocardial strain imaging is one of the most important new technologies in recent years, which provides information on myocardial deformation and has the potential to detect subtle changes in myocardial function that may not be apparent with conventional imaging methods.

The work written by Zlibut et al. provides a comprehensive overview of the available data on the role of CMR in evaluating myocardial strain and biomechanics—highlighting its potential as a valuable tool for early detection, diagnosis, and management of cardiovascular diseases [13]. The authors highlight two specific CMR techniques, tissue-tagging (TT-CMR) and feature-tracking CMR (FT-CMR), that have been shown to accurately determine deformation parameters and functional dynamic geometry parameters. The authors point out that these techniques have been studied extensively in ischemic heart disease and primary myocardial illnesses and have shown utility in prognostic prediction in various cardiovascular patients. They also discuss the recent emergence of fast strain-encoded imaging CMR-derived myocardial strain as a potentially superior method for measuring myocardial strain due to its accuracy and reduced acquisition time. However, it should be acknowledged that more studies need to be carried out to establish its clinical impact.

Michler et al. compared two methods, the conventional contour surface method (KfM) and the pixel-based evaluation method (PbM), for determining left ventricular function parameters in CMR imaging [14]. The KfM includes the papillary muscle as part of the left ventricular volume, leading to a systematic error in the calculation of the left ventricular ejection fraction (LVEF). The PbM, on the other hand, excludes the papillary muscle volume and provides more accurate results. The study analyzed 191 CMR image data sets and found that the PbM showed a negative difference for end-diastolic volume (EDV), a negative difference for end-systolic volume (ESV), and a positive difference for LVEF compared to the KfM. There was no difference in stroke volume (SV). The PbM also had a mean papillary muscle volume of 14.2 mL and took an average of 2:02 min for evaluation. The authors conclude that the PbM is an easy and fast method for determining left ventricular cardiac function and provides comparable results to the established KfM while omitting the papillary muscles. This can have a significant influence on therapy decisions, as the LVEF may be 6% higher with the PbM.

A study conducted by Ueda et al. explored the use of FT-CMR and self-gated magnetic resonance cine imaging to evaluate cardiac function in a young mouse model of Duchenne muscular dystrophy (mdx) [15]. The results show that the LVEF was significantly lower in the mdx group compared to that in the control group at both time points. Strain analysis also revealed significantly lower strain values in mdx mice, with exception of the longitudinal strain of the four-chamber view. This study concludes that strain analysis with feature tracking and self-gated magnetic resonance cine imaging is useful for assessing cardiac function in young mdx mice.

## 6. Summary

This Special Issue of “Advances in Cardiovascular Magnetic Resonance” highlights the role of CMR imaging in improving the diagnosis, management, and prognosis of various cardiovascular diseases. From its ability to detect LGE in patients with IDCM to its role in diagnosing Loeffler endocarditis as well as its potential for predicting LVR following PPCI in AAMI patients, CMR imaging is proving to be a valuable diagnostic tool.

Finally, the comprehensive review of the available data on the role of CMR in evaluating myocardial strain and biomechanics underscores the potential of these techniques in providing a more complete assessment of cardiac function and mechanics, as well as in detecting subtle changes in myocardial strain and deformation in various cardiovascular conditions.

As the field of cardiovascular imaging continues to advance, CMR imaging is poised to play an increasingly pivotal role in detecting, diagnosing, and managing cardiovascular diseases.
